# Electrical Impedance of Upper Limb Enables Robust Wearable Identity Recognition against Variation in Finger Placement and Environmental Factors

**DOI:** 10.3390/bios11100398

**Published:** 2021-10-16

**Authors:** Hyung Wook Noh, Joo Yong Sim, Chang-Geun Ahn, Yunseo Ku

**Affiliations:** 1 Bio-Medical IT Convergence Research Department, Electronics and Telecommunications Research Institute, Daejeon 34129, Korea; happy05@etri.re.kr (H.W.N.); cgahn@etri.re.kr (C.-G.A.); 2 Department of Biomedical Engineering, College of Medicine, Chungnam National University, Daejeon 35015, Korea; 3 Department of Mechanical Systems Engineering, Sookmyung Women’s University, Seoul 04310, Korea; jysim@sookmyung.ac.kr

**Keywords:** wearable biometrics system, electrical impedance, upper limb impedance, ratiometric

## Abstract

Most biometric authentication technologies commercialized in various fields mainly rely on acquired images of structural information, such as fingerprints, irises, and faces. However, bio-recognition techniques using these existing physical features are always at risk of template forgery threats, such as fake fingerprints. Due to the risk of theft and duplication, studies have recently been attempted using the internal structure and biological characteristics of the human body, including our previous works on the ratiometric biological impedance feature. However, one may still question its accuracy in real-life use due to the artifacts from sensing position variability and electrode–skin interfacing noise. Moreover, since the finger possesses more severe thermoregulatory vasomotion and large variability in the tissue properties than the core of the body, it is necessary to mitigate the harsh changes occurring at the peripheral extremities of the human body. To address these challenges, we propose a biometric authentication method through robust feature extraction from the upper-limb impedance acquired based on a portable wearable device. In this work, we show that the upper limb impedance features obtained from wearable devices are robust against undesirable factors such as finger placement deviations and day-to-day physiological changes, along with ratiometric impedance features. Overall, our upper-limb impedance-based analysis in a dataset of 1627 measurement from 33 subjects lowered the classification error rate from 22.38% to 4.3% (by a factor of 5), and further down to 2.4% (by a factor of 9) when combined with the ratiometric features.

## 1. Introduction

Biometric authentication refers to a security process that verifies a user’s identity through unique physical and behavioral characteristics of the human body, such as fingerprint, iris, voice, and gait [1]. In a technology-driven, connected modern society, complex password requirements pose significant inconvenience to an increasing number of users [2]. Biometric authentication, unlike passwords, tokens, or access cards, verifies personal identity without the need for users to remember passwords or carry physical objects. Therefore, it can be used to increase user convenience and efficiency for the numerous online access and mobile transactions that we use today [3]. Biometric authentication technologies, which are based on the structural features of acquired images such as fingerprint, iris, and facial recognition, are most commonly used in the biometric field due to its advantages, such as convenience and simplicity, and recently released smartphones or tablet computers as a form of IT systems mainly include these user recognition features [4]. However, these image-based biometrics can be easily stolen or spoofed, and their anti-counterfeiting performance can also be threatened [5]. For example, various fingerprint scanners that ensure security are widely used, but attacks using silicone, gelatin or Play-Doh counterfeits made from users’ fingerprints are widespread, and this poses a considerable threat [6]. It has also been reported that iris-based authentication systems are vulnerable to spoofing via photographic irises and well-crafted colored lenses [7]. In particular, unlike passwords that can be modified or reissued, fingerprints, faces, or irises are exposed to permanent threats to spoofing once the template is stolen. To overcome these challenges and increase the level of security, biometric studies based on bio-signals such as electrocardiograms [8], electromyograms [9], electrooculograms [10], and electroencephalograms [11] are increasing. However, these bio-signals are not robust enough to serve alone as biometric traits because they can be dependent on the user’s physiological state, exercise, or emotional state [12,13,14]. While liveness detection is also an active area of research to mitigate the vulnerability of biometric systems, various spoofing attacks still exist to defeat these authentication solutions [15]. Attackers can easily circumvent the liveness detection by developing specific spoofing artifacts to trick biometric authentication, which can be faced with a significant loophole [16].

Bioelectrical impedance measurement is a fast, non-invasive technique that measures the physiological state of living tissue and responds less sensitively to emotional conditions [17]. Bioelectrical impedance analysis estimates body compartments through a mechanism of resistance and reactance [18]. The electrical impedance encompasses information about the presence of specific tissue types such as blood, muscle, and bone, and anatomical composition inside the measurement site [19,20,21]. Therefore, bioelectrical impedance can be used as a biomarker for diagnosing physiological conditions [22,23]. Each individual has significant electrical impedance differences due to these complex and diverse internal tissues and anatomical compositions of the human body [24,25,26]. Therefore, bioimpedance-based analysis of complex tissues can provide unique mechanisms to distinguish people [27]. Furthermore, since the electrical impedance of body tissues depends on the alternating current signal frequency [28,29,30], multiple frequencies for impedance can be used to enhance identification performance. Nevertheless, bioelectrical impedance has not been realized as commercial identity authentication technology due to its low reproducibility caused by changes in various factors such as body fat, skin moisture, and blood vessel expansion due to body temperature [31]. Skin blood flow and body temperature changes, as well as skin electrolyte accumulation are known to largely affect impedance measurements [32,33,34]. Previous studies have reported that various physiological conditions such as eating, exercise, fluid retention, and obesity can affect impedance measurements, and also found that body temperature is inversely related to changes in impedance [31,32,35,36,37,38]. Therefore, the susceptibility of impedance to body temperature or other physiological changes may limit its use as robust biometric authentication. To overcome these limitations, our previous work [39] proposed a novel ratiometric method to obtain reliable features from multi-channel impedance of finger combinations. The ratiometric method assumes that when the electrical properties of the finger vary, the electrical properties of all finger components change at the same proportion, and thereby the method obtains ratiometric features by obtaining the proportion between the impedances of one particular finger pair and another. The ratiometric feature was shown to improve the classification accuracy from 70.7% to 94.1% and equal error rate (EER) down to 3.0%. Nevertheless, the biometric authentication based on biological impedance is still less accurate for commercialization compared to traditional methods such as fingerprint (EER < 0.1% [40]) and face recognition (accuracy > 99% [41]).

Biological impedance measurement is not limited to hands or fingers, but can be applied to any part of the body, which opens up new possibilities to increase convenience by configuring it in various forms, such as wearables, and to increase accuracy by measuring in various body parts. Nonetheless, there has not been much research on comparing the performance of biometric authentication when measuring body impedance in diverse body parts. A notable point is that the degree to which biological impedance changes due to physiological and environmental factors varies on different parts of the body. For instance, changes in body temperature due to the ambient temperature can differ in each body part [42,43]. Previous studies reported that the temperature variations in the fingers, hands, and feet are greater than in other core parts of the body (e.g., head and arms) when the subjects were exposed to a cold environment [43,44]. Arens et al. showed that the hands and feet were considerably colder than the head and arms in a cold environment, and thus vasoconstriction was the strongest [45]. These different physiological changes in distinct body parts have been shown to affect the biological impedance measurement [46,47,48]. Therefore, the choice of body part is expected to influence the biometric authentication performance (e.g., the peripheral end of extremities such as fingers, or parts of the body close to the core such as the upper limb).

In order to exploit the features of these different parts of the body, it is necessary to devise methods to form electrode contacts and arrange effective electrical paths. A promising strategy for collecting signals in different body parts is using wearable devices [49]. Existing product types of wearable devices span across smart watches, smart bracelets, smartphones, smart glasses, tablets and PCs. With diverse form factors of wearables and mobiles, identity recognition becomes increasingly significant to provide personalized services of Internet of Things (IoT) devices which provide convenience and accessibility to people’s lives [50]. Security becomes increasingly important with the use of a large number of IoT devices that require interaction between smart devices and consumers. Biometrics provide improvements in the usability and security of IoT and can play an important role in securing emerging IoT devices to address security challenges. For example, a wearable biometric authentication device through integration with a smartphone-based network substantially expands the range of service applications [51]. Implementing biometrics in a wearable device has also been shown to increase the collectability and convenience for practical biometric systems [52,53,54,55]. These wearable devices have the advantage of being lightweight, portable, and easy to use; however, signal distortions and artifacts are a challenge to be overcome. In comparison to standalone immobile systems, biometric sensing in mobile and wearable devices undergoes large variation in user behavior and changes in environmental factors [51], which can cause motion/skin–electrode artifacts. Therefore, researchers have been conducting extensive studies to reduce these artifacts in wearable measurement devices [56,57]. For instance, researchers have examined how the skin–electrode interface in wearables contributes to the signal noises for electrodes [58]. However, previous studies mainly focused on the skin–electrode contact interfaces, but few have shown a method to overcome changes in the relative position of the electrode and the skin. Motion artifacts as well as inconsistent placement of the hands and fingers often arise from the interface due to the relative motion of electrodes to the skin, which ultimately limit the repeatability and inevitably affect the performance [59]. Thus, despite the wearable form factors applied to various body parts widening the opportunities of bioimpedance-based identity recognition, they need to address challenges resulting from uncontrollable harsh operating conditions and large variability.

In this study, to address the aforementioned challenges, we propose a wearable biometric authentication system that can obtain robust impedance features of fingers as well as upper limbs and verify its applicability through the evaluation of data acquired from our proposed wearable device. The hardware configuration and the principle allow us to acquire the upper limb impedance that includes arms and shoulders through the electrical path between the right-hand fingers and the left wrist. We observe that the upper limb impedance features are less variable than the finger impedance by external environmental effects such as skin and air temperatures and environmental humidity [60,61,62] thanks to physiological homeostasis. We investigate that the upper limb impedance extraction can eliminate the artifacts of finger placement variation, which can occur more frequently in wearable device measurements [63,64]. To assess the reproducibility and user classification capability of upper-limb impedance features, we conduct a user classification test of 33 subjects and analyze the performance of various machine learning and CNN-based deep learning models. Additionally, we investigate whether the upper limb impedance features have a synergistic effect with the ratiometric feature of fingers to improve the classification accuracy. Our proposed wearable device, integrated with wireless communication, demonstrates the potential of a single portable biometrics technology, offering solid security with advantages of collectability, performance, and convenience. Our study is expected to provide a guideline for developing biological-impedance-based biometric systems which are extremely secure and show high performance in wearable or mobile applications.

## 2. Results and Discussion

### 2.1. Impedance-Based Wireless Wearable Identity Recognition System

**Figure 1:**
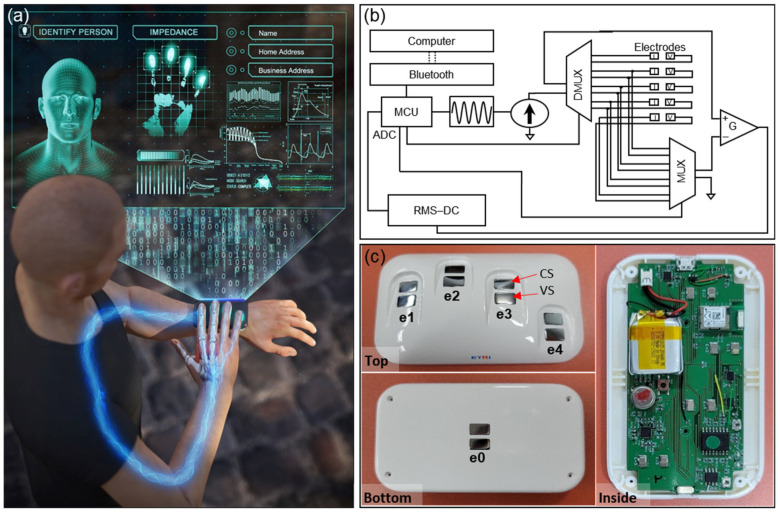
Schematic of wireless wearable electric impedance spectrum identity authentication system. (**a**) Concept of wearable identity recognition system which employs the electrical transfer characteristics through fingers and upper limbs. (**b**) Schematic of the wearable impedance-based identity authentication system. The frequency-modulated sinusoidal current is transmitted sequentially to the electrode pairs by the driving of the multiplexer and demultiplexer, converted to a DC signal by the RMS-DC converter, and then digitized by the ADC of the microcontroller. (**c**) Our developed impedance-based wireless identity authentication module. Numbers 1 to 4 (*e*_1_*~e*_4_) are assigned in order from index to little finger electrodes, and number 0 (*e*_0_) is assigned to left wrist electrode mounted under the device. Separate current sourcing (CS) and voltage sensing (VS) electrode pairs for applying four-point electrode measurements were mounted on the device.

Our proposed approach evaluates the characteristics of the human body and tissues by transmitting electrical signals to the body, especially the fingers and upper limbs to acquire electrical response characteristics at various frequencies. Figure 1 shows the proposed identity recognition system that obtains the electrical characteristics of the right fingers in contact with the device located on the left wrist, as well as the upper limb impedance measured by using the electrode mounted under the device. The schematic diagram of our wearable impedance measurement system is shown in Figure 1b. Via Bluetooth, we enabled wireless control of impedance measurement circuits, and the measurement circuits consisted of a multiplexer, a microcontroller capable of switching each pair of measuring electrodes, a waveform generator and a constant current source. A battery was mounted inside the device, and a battery protection circuit was also implemented to ensure safe charging. Figure 1c shows the exterior (top, bottom) and interior of the wearable device incorporating a grooved design for electrodes and finger guides, and the printed circuit board including the battery, respectively. The four electrodes mounted on the upper side of the device were for contacting the right fingers, and the other electrode mounted under the device was for contacting the left wrist. Impedance measurement was performed for a total of 10 combinations by selecting two of five electrodes by the multiplexer. The impedance of 10 combinations was measured in sequence by electrically switching the five electrodes mounted on the device. Among the selection of 10 electrode combinations, the impedance including the upper limb was obtained from four combinations (*e*_0_, *e_i_*), where *i* = 1, 2, 3, 4, in which one electrode contacting the wrist was selected. Then, the impedances of the pairs of right fingers were obtained through the combinations, (*e*_1_, *e*_2_), (*e*_1_, *e*_3_), (*e*_1_, *e*_4_), (*e*_2_, *e*_3_), (*e*_2_, *e*_4_), and (*e*_3_, *e*_4_), of pairs of electrodes mounted on the device. The electrical impedance spectrum for each combination was measured in the frequency range of 20 kHz to 500 kHz. A constant source current was passed through the two current source (CS) electrodes, and the voltage across the CS electrodes was measured by voltage sensing (VS) electrodes located between the electrical paths. The voltage measured at the vs. electrode pair was amplified by the differential Amplifier (G). The four-point measurement design shown in Figure 1c improved reproducibility by minimizing the effect of contact resistance for every measurement. The data measured using the wearable device were directly transmitted to a laptop computer paired with the device via Bluetooth communication.

### 2.2. The Principle of Proposed Upper Limb Feature Extraction

**Figure 2:**
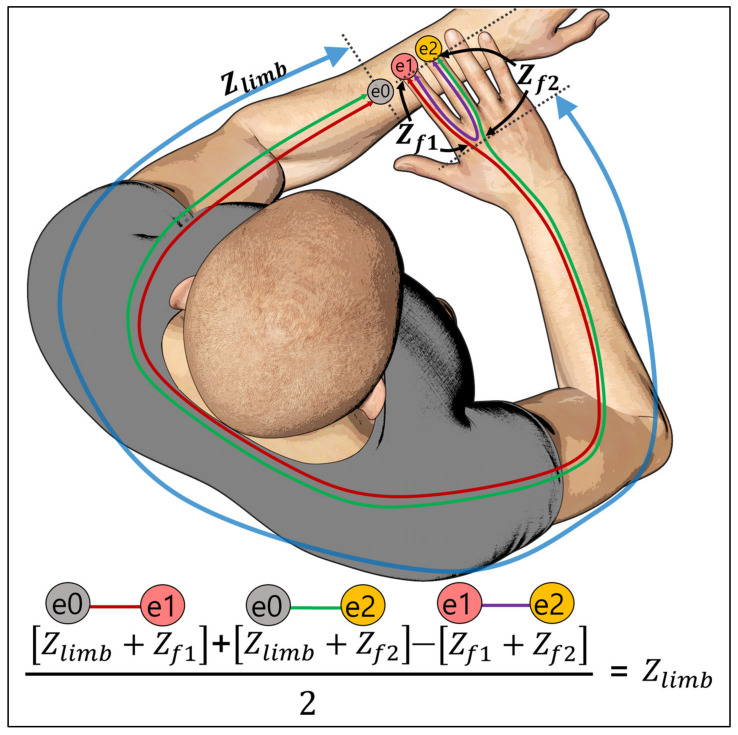
Principle of peripheral variation removal. The pure upper limb impedance can be obtained by subtracting the impedance of a pair of fingers (e.g., (*e*_1_, *e*_2_)) from the sum of the impedances from electrode *e*_0_ (e.g., (*e*_0_, *e*_1_) + (*e*_0_, *e*_2_)).

Our proposed method extracts robust impedance features of upper limbs by subtracting finger impedance to overcome large variability of the impedance of body extremities such as the hand or finger due to changes in external environments (e.g., temperature, humidity). To enhance the reproducibility and utilize the relatively steady characteristics of the upper limb, we devised a method to extract features that are robust to undesirable changes by excluding the finger impedance from the measurement data. For data acquisition in our wearable device, a user places four fingers on the electrodes mounted on the device on the user’s left wrist, as shown in Figure 2. In this measurement mechanism, when the electrode ‘*e*_1′_ is selected as a pair for the ‘*e*_0′_ (*e*_0_, *e*_1_), the impedance of the upper limb (Z*_limb_*) including finger 1 (Z*_f_*_1_) is obtained, and when ‘*e*_2′_ is selected, that is, (*e*_0_, *e*_2_), the impedance of the upper limb (Z*_limb_*) including finger 2 (Z*_f_*_2_) is obtained. To obtain a pure upper limb impedance, we first summed the impedance (Z*_limb_* + Z*_f_*_1_) measured from (*e*_0_, *e*_1_) and the impedance (Z*_limb_* + Z*_f_*_2_) measured from (*e*_0_, *e*_2_). Then, we subtracted the measured impedance (Z*_f_*_1_ + Z*_f_*_1_) from (*e*_1_, *e*_2_) to remove the two finger impedances (Z*_f_*_1_, Z*_f_*_2_) included in the summation. By dividing the resulting value by half, the pure upper limb impedance was obtained.

### 2.3. Robust Features in Upper Limb against Thermal Environments

**Figure 3:**
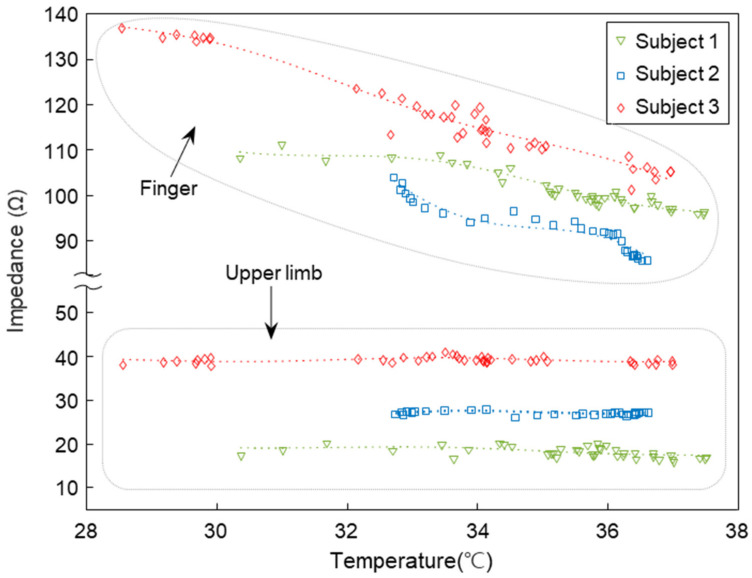
Impedance variation according to temperature. Changes in impedance of fingers and upper limbs acquired by three subjects (Subject 1~Subject 3) according to temperature. The impedance in *Y*-axis is an average in the frequency range from 20 kHz to 500 kHz.

As explained above for the low reproducibility of biological impedance, our body impedance varies greatly depending on external environmental factors, such as temperature, and these effects can vary dependent on the region of the body [65]. The core temperature is relatively homogeneous throughout the trunk, while the temperature of peripheral tissues, such as the hands or fingers, varies greatly with environmental factors and thermoregulatory vasomotion [42]. For those reasons, we examined and compared the effect of external temperature changes on the impedance of upper limbs and fingers. We conducted tests in three healthy adults in a temperature-controlled laboratory. Room temperature was controlled in the range of 32.0~18.0 °C, and the test was performed while cooling from a high temperature to a low temperature. The temperature of the body was measured for each test, and the hand temperature changed as the room temperature decreased, and the armpit temperature was constant in the range of 36.8~37.2 °C. To examine the impedance changes with temperature, we used the average of all impedance values obtained over the frequency range of 20 kHz to 500 kHz. The finger impedance of the three subjects was inversely proportional to the measured hand temperature, as shown in Figure 3, whereas the upper limb impedance features extracted from the same experiment stayed constant with environmental temperature changes. These results indicate that changes in external temperature are an important factor influencing impedance measurements, and that upper limb impedance is relatively robust to these changes and can be used as a feature to improve reproducibility.

### 2.4. Enhanced Upper Limb Features Reducing Undesirable Variation

There are several other factors, such as inherent changes of normal physiology, that influence biological impedance, as reported in other previous studies mentioned above. The effects of these factors may also differ between the fingers and the upper limb. To examine the effects of these other factors and to compare the differences in effects on the fingers and upper limbs, day-by-day impedance measurements were performed for five subjects at the same temperature over three independent days.

**Figure 4:**
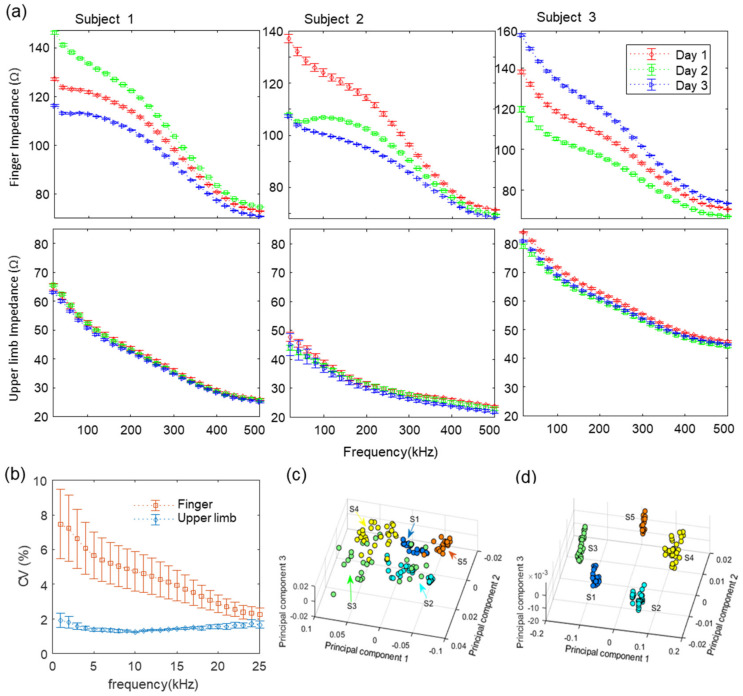
Comparison of temporal variation in finger and upper limb impedance features. The impedance of fingers and upper limbs were obtained over three independent days. (**a**) Finger (above) and upper limb (below) impedances of three representative subjects measured over three independent days for assessing temporal variation. The error bars in the figure indicate the relative standard deviation (*n* = 10). (**b**) Coefficients of variation of finger impedance and upper limb impedance. (**c**) Scatter plot of the first three principal components of the finger impedance from principal component analysis. (**d**) Scatter plot of the first three principal components of the upper limb impedance from principal component analysis.

Figure 4a shows the change in finger impedance (above) measured and upper limb impedance (below) extracted from the corresponding each finger impedance from each date for three subjects. The impedance data was measured for each subject in total 10 times per day, and the data were expressed by mean and standard deviation. It can be seen that the impedance curves of fingers for each subject change according to the date of measurement. We speculate that these changes were due to various physiological factors, such as changes in internal and external moisture, blood flow or vessel expansion. These changes in fingers can be more sensitive to physiological changes related to blood flow because normalizing hand blood vessels to the skin surface area is 4 to 5 times larger than the rest of the body [66]. The graphs located below Figure 4a show the upper limb impedance extracted from the data measured for each subject. The upper limb impedance curves of the respective state for each subject were mostly overlapped and coincident in the same pattern, in contrast to the finger impedance, which varied by measurement dates. In addition, to better understand the direct effect, we compared the upper limb impedance before and after finger removal through a compensation process, to allow the readers to better understand the effect directly. Therefore, we placed the two figures of upper limb impedance (a) before and (b) after finger removal for the three subjects together and comparing them side-by-side in Supplementary Figure S1. Here, it can be seen that the upper limb impedance after removing the finger impedance improves the reproducibility compared to before the compensation process. To statistically compare the variation of the quantitative characteristics of finger and upper limb impedance by date, we analyzed each data point using the coefficient of variation [67] (*CV*, standard deviation (*s*) divided by the mean ($\bar{x}$)), as shown in the following equation:

(1) $$CV=\frac{s}{\bar{x}}$$

Figure 4b shows the data variability of finger and upper limb impedance by date. The data, which were repeatedly measured 10 times per day, were averaged for each day, and *CV* was calculated for each subject. Then, the *CV*s calculated for each subject were expressed as the mean and standard deviation. The data variability of the finger was greater than the upper limb at all measurement frequencies, and this difference in particular was greater for the lower frequency band. To visualize and compare the distinguishability of the electrical impedance of fingers and upper limbs, principal component analysis was performed on each corresponding data point, and the first three principal components of the spectra for both features are displayed in Figure 4c,d respectively. Principal components were more clearly clustered in upper limb features than the finger impedance. The ratiometric method, proposed in our previous study [39], was applied to the data measured in this study. The ratiometric features extracted from each subject’s finger impedance showed improved reproducibility, as shown in Supplementary Figure S2.

### 2.5. Robust Upper Limb Features against Finger Placement Variation

**Figure 5:**
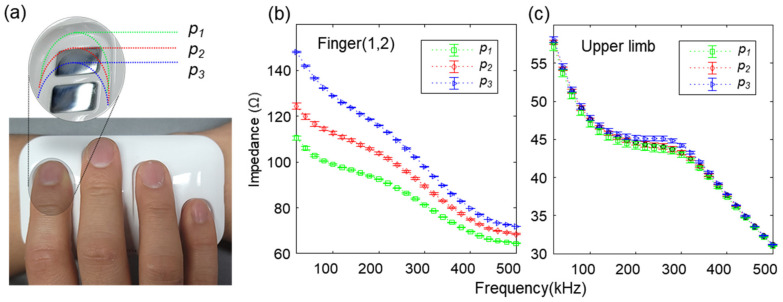
Impedance variation of finger and upper limb according to finger placement changes on the electrode. (**a**) Changes in the contact position (*P*_1_*~P*_3_) of the subject’s index finger. (**b**) Variation of finger impedance according to the three electrode contact positions. (**c**) Variation of upper limb impedance according to the three electrode contact positions. The data are presented as mean ± standard deviation (*n* = 10).

Most of the undesired artifacts by misplacement are associated with the skin contact position with the electrode, resulting in changes in the electrical path, but these effects can be eliminated, especially in the upper limb feature. Note that we removed the contact resistance between skin and electrode by using four-point measurement, as described in our previous work [39]. Despite the fact that eliminating contact resistance helps to improve robustness against changes in the finger contact condition, the finger contact changes can still alter the electrical path between two measurement points. To examine this effect, we observed the variation in finger impedance by changing index finger placement positions (*P*_1_*~P*_3_) with respect to the electrode, as shown in Figure 5a, and compared this with the upper limb impedance. The data were measured 10 times per position and displayed as mean and standard deviation. The measured data for the three extreme placement position variations (*P*_1_*~P*_3_) showed significant finger impedance variation, as shown in Figure 5b. We speculate that the variability of finger placement positions caused changes in the length of current flow and the position of voltage sensing in the electrical path. In addition, the contact area between the finger and electrode varies depending on the finger placement position, which changes the electrical path. Even in the case of the ratiometric method proposed to improve reproducibility in our previous work [39], it is vulnerable to signal distortion due to measurement position changes.

The ratiometric method improves reproducibility regardless of changes in the external environment; however, the ratiometric features can still be at a risk of experiencing artifacts due to different finger placement position. For example, when only a specific single finger position can be changed, the reproducibility of the ratiometric feature is inevitably lowered. Supplementary Figure S3 shows the positional (*P*_1_*~P*_3_) variability of three ratiometric features extracted from measured impedance data. However, the artifacts intruded from finger–electrode interfaces can be removed in the upper limb impedance through the mechanism described in Figure 2. Figure 5c shows that the upper limb impedance features are highly reproducible and relatively robust against the variation of each finger position (*P*_1_*~P*_3_). The experimental results indicate that the upper limb features extracted by using the wearable device are reliable regardless of the influence of finger placement artifacts, which are more likely to occur on measurements in wearable devices than a standalone, immobile system.

### 2.6. Improved Identity Recognition Performance via Upper Limb Features

**Figure 6:**
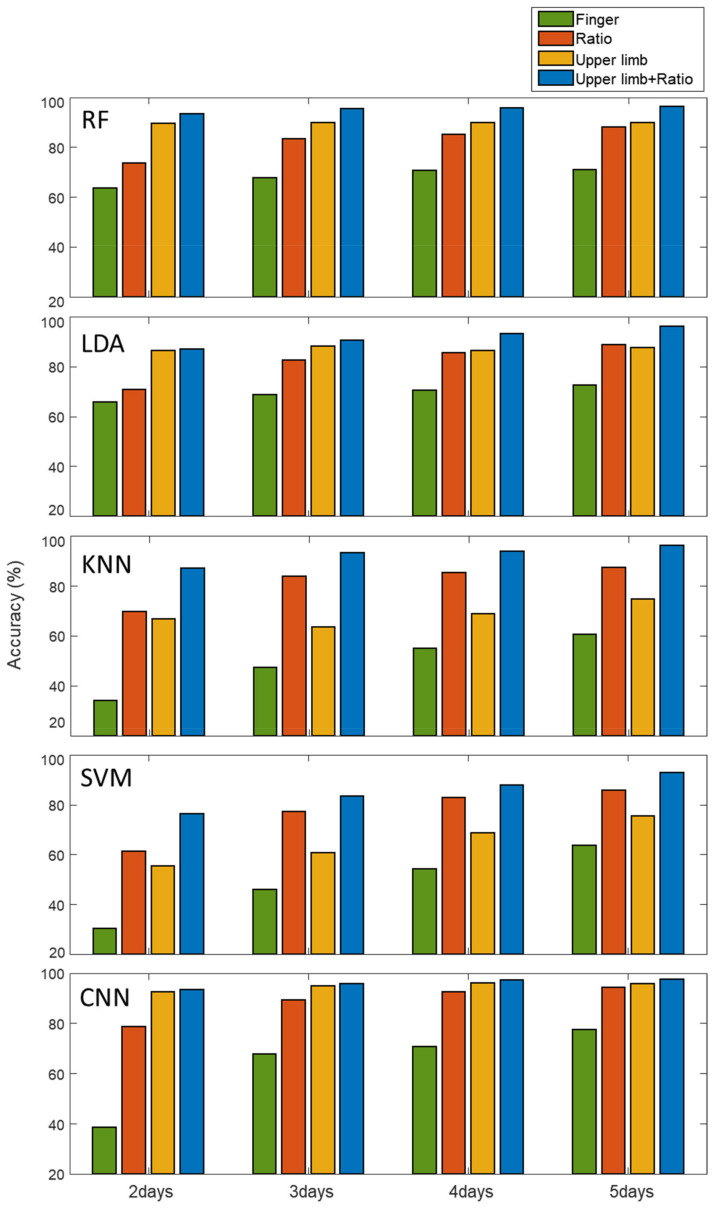
Comparison of identification accuracies according to the types of features used in the five different classification models.

To develop a predictive model that makes accurate predictions on new data, unseen during training, we needed a sophisticated technique, which carefully constructed a training dataset that models were training on, as well as a test dataset without any data leakage from the training dataset [68,69]. Since biological impedance can vary from day to day due to physiological state changes, the data measured on the same day from one subject tend to be more similar than data measured on the other days. Therefore, if data collected on the same date are present in both the train and test datasets, it is prone to data leakage and overfitting problems. Therefore, we mutually excluded the data measured on the same date from the train and test dataset by dividing each subject’s data into subgroups and labelling each data point from one to five according to the order of the measurement dates, which was used for cross validation. Through this process, the measured data for each date were predicted by the model, learned from the data obtained on different dates. The identification performance based on biological impedances, including the upper limb, was evaluated from data collected from 33 subjects. Subjects participated in five days of sessions at one-week intervals and provided 8~10 measurements for each session within an hour per day. For the identification of individuals, we applied the discriminative machine learning models of random forest (RF), linear discriminative analysis (LDA), K-nearest neighbor (KNN) and support vector machine (SVM), and additionally applied a convolutional neural network (CNN, Supplementary Figure S4) for deep learning analysis. For the machine learning models, the feature vectors from each channel consisting of 25 samples were concatenated, merged into a single feature vector, and fed into the classifiers. For CNN models, each one-dimensional feature vector was used as a form of each channel in 1D input and fed into the model. In particular, we assessed the classification accuracy with the accumulated number of data-acquisition days from 2 to 5 days using n-fold (n: number of measurement days) cross validation. Figure 6 shows an ablation study that compares the performance depending on the types of features as follows; (i) the raw finger impedance, (ii) ratiometric features, (iii) upper limb impedance only, and (iv) ratiometric features together with upper limb impedance. Accuracies derived from all the models were significantly improved when using upper limb impedance, compared to using raw finger impedance. In addition, the performance of the RF, LDA, and CNN was better with upper limb impedance than with ratiometric features. In contrast, in the KNN and SVM models, the ratiometric-feature-based models performed better than the models with upper limb impedance. Overall, using both the ratiometric features and the upper limb features (iv) outperformed the other three (i–iii). In all classification models, performance gradually improved as the number of measurement days increased, especially when using ratiometric features. We speculate that as the measurement dates increased, the training dataset reduced the bias of a specific day, making the model learn traits that were invariant to the measurement dates and more robust against day-to-day variation.

Relatively, the accuracy of models based on the upper limb feature showed few changes with the number of measurement days. This result indicates that the upper limb impedance is rather robust against day-to-day variations caused by several aforementioned undesirable factors. Note that the performance of the ratiometric-based models was significantly degraded when using datasets with only few measurement days (e.g., 2 or 3 days). However, we observed a significant improvement by using the upper limb features, and decent classification accuracy could be obtained even with upper limb features alone in RF, LDA and CNN models. In particular, the classification accuracy yielded 92.58% accuracy when only 2 days of datasets were used in the upper-limb feature-based CNN model. This result shows that the upper-limb feature-based analysis can achieve a reliable performance with fewer data acquisition days, while the ratiometric features require a relatively large number of measurement days to achieve improved accuracy. Thus, the ratiometric features exhibit a synergistic effect with upper limb features in improving classification performance. Using a total of 1627 data points for 5 days, we achieved the highest classification accuracy of 97.6% by using both upper limb and ratiometric features with the CNN model while other models showed accuracy of over 92%, as shown in Table 1. Taken together, the upper limb features improved the classification performance significantly, and helped to provide reliable and robust identity authentication systems for wearable applications.

### 2.7. Comparison of Performance with Increasing Date of Measurement and Type of Feature Used

**Figure 7:**
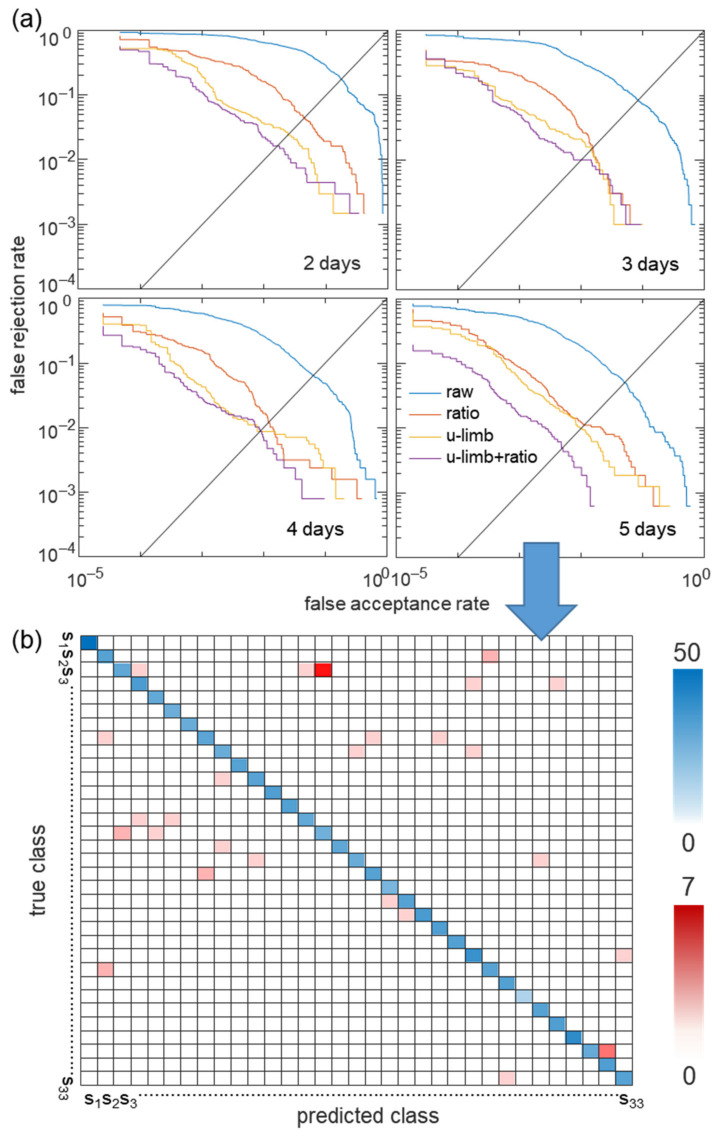
Individual identification performance with increasing date of measurement and according to four different types of features used ((i) the raw finger impedance, (ii) ratiometric features, (iii) upper limb impedance only and (iv) ratiometric features together with upper limb features). (**a**) ROC curves for each selected feature with increasing number of measurement dates. (**b**) Confusion matrix of the CNN classifier among 33 subjects in the case of 5 measurement days using upper limb features together with ratiometric features, where the intensity in the blue and red boxes indicates the number of correct and incorrect predictions, respectively.

The accuracy of our discriminant classifiers was evaluated based on closed-set identification, allowing a given observation to be assigned to the class with the largest latent variable or best posterior probability to distinguish one from the others. Thus, this can cause errors of assigning a class that provides the highest probability score for unregistered users. However, in biometrics, if the acquired feature vector does not provide a sufficiently large posterior probability, the algorithm must be able to recognize an unregistered user and reject the case. Using the threshold of the latent variables of the classifier, the measured data were allowed to be assigned to the classes only if the posterior probability of the data was greater than the threshold. This method enables recognizing an unknown user who was not enrolled in training process. By changing the threshold in the CNN model that yielded the highest accuracy, we evaluated the sensitivity (1—false acceptance rate, FAR) and specificity (1—false rejection rate, FRR) for the receiver operating characteristic (ROC) curve. The ROC curves of the classification results based on the number of measurement days (2, 3, 4, and 5) for each feature selection in the CNN model are shown in Figure 7. The horizontal axis represents the false acceptance rate, and the vertical axis represents the false rejection rate, in which the more adjacent to the lower left corner the curve is, the better the performance is. In the case of a dataset with a small number of measurement days, such as 2 or 3 days, the ROC curve of the upper limb feature was significantly better than that of the ratiometric feature. As the number of measurement days increased, the ROC curve of the ratiometric feature improved. Additionally, using both upper limb and ratiometric features substantially improved performance, as shown in the ROC curves for all cases. The ROC curve was used to evaluate the area under the ROC curve (AUC) and an equal error rate (EER) at which FAR was equal to FRR. In the case of datasets of 5 days, the upper limb feature reduced the classification error rate from 22.38% to 4.3% compared to the finger impedance (raw data), and further reduced it to 2.4% by combining it with the ratiometric feature, resulting in an AUC of 0.9999 and an EER of 0.49%. Figure 7b shows a confusion matrix of 33 subjects using both upper limb and ratiometric features in CNN for the dataset of 5 days. The intensity of the color of the blue box represents an accurate prediction, and that of the red box represents a false prediction. ‘S_1_, S_2_, S_3_, ⋯ S_33′_ represents each subject class. Most of the predictions are located in the blue-colored diagonal matrix.

Table 1 shows the accuracy, EER, and AUC in the tested CNN according to feature selections (finger impedance, ratiometric, and upper limb features). The CNN model produced the highest performance of accuracy, EER, and AUC in all feature selections. In RF, LDA and CNN models, the upper-limb feature was a salient feature improving the classification accuracy, while in KNN and SVM models, the ratiometric feature achieved better results. All accuracy, EER, and AUC values derived from each model were significantly improved when using the upper limb impedance including ratiometric features than only using the finger impedance (raw data).

### 2.8. Scalability Analysis

We performed additional experimental analysis of scalability of our system to validate the practical applicability and utility of biometric information extracted in this study. To examine whether our system has the potential to manage large-scale classification, we analyzed the change in classification accuracy with a change in the number of subjects. The number of subjects was increased by five from a minimum of five subjects, and each dataset was randomly selected from a total of 33 subjects. Each classification accuracy was extracted using the RF model, and the process was repeated 10 times to extract the average accuracy and standard deviation. The analysis result, presented as boxplots in Supplementary Figure S5, shows that the accuracy decreased as the number of selected subjects increased. The decrease in classification accuracy gradually slowed as the number of subjects increased, which led us to hypothesize the decay of the accuracy prediction curve as a log-scale exponential function. The prediction curve converged to 97.38%, which is reasonable accuracy for practical biometric authentication applications. In future studies, we will validate our system in a large population to compare our method with conventional biometrics.

## 3. Conclusions

Our research is meaningful as a biometric authentication technology implemented in a wearable environment with convenience that can solve the risk of duplication of traditional image-based technologies and the existing limitations of the lack of reproducibility. The research into anti-spoofing technologies, such as liveness detection, is active with increasing attempts to forge biometric systems, but biometric systems are still vulnerable to sophisticated spoofing attacks [70,71,72,73,74]. Our devised method, which analyzes non-image-based characteristics, can provide a higher level of security that cannot be replicated, as spoofing attempts must mimic the user’s anatomical and biomaterial properties to be authorized. In order to evaluate the effectiveness of a wide range of practical applications such as convenient and continuous identity authentication based on a wearable environment, the impedance measurement system developed in previous research was optimized and miniaturized. We speculate that such wearable biometric authentication can be combined with various technologies to improve not only performance, but also human comfort and convenience. In this study, the applicability to the actual wearable biometric authentication system was verified by evaluating the performance according to the increase in the number of measurement days as well as the selection of various features, including upper limb impedance acquired from the developed wearable device. Our results provide evidence that upper limb impedance can be used to uniquely identify individuals. It has been shown that the upper limb impedance features improved the classification accuracy, and also had a synergistic effect when used with the ratiometric features, which greatly improved the classification accuracy and significantly reduced the error rate. The results that showed a significant improvement in performance using upper limb features demonstrated the importance of enhancing reproducibility through the removal of finger impedance including artifacts such as electrode–skin interfacing noise or physiological changes caused by environmental influences. The developed wireless wearable device has proven its potential as a portable identity authentication technology and has shown that it can provide reliable security as well as performance and convenience. It is expected that the proposed method based on upper limb impedance features will be able to provide an innovative authentication technology against spoofing attacks in a wearable environment.

## 4. Materials and Methods

### 4.1. Impedance-Based Wireless Wearable Identity Recognition System

Electrical signals transmitted through the fingers and upper limbs include relevant biomaterials, bioelectrical properties, and anatomical information in the electrical pathway. To implement identity authentication based on the electrical characteristics of the upper limb and fingers, we designed a wearable device that detects electrical impedance by applying a modulated sinusoidal constant current. Since the biometric authentication-based system in our study is a wearable device, it should be compact in size with low power and provide stable performance to obtain reliable data. All the components were selected to satisfy the form factor of our device size, with a maximum size of 10(W) × 10(L) × 5(H) mm^3^ or less. In addition, to achieve reliable performance for classification, it is important to obtain a salient and stable feature with a high resolution of the acquired signal by using a sensing unit with high input impedance. The dimensions of the device are 8(W) × 5(L) × 1.7(H) cm^3^. Our device included an MCU (Atmega328p, Microchip Technology, Chandler, AZ, USA), a programmable waveform generator (AD9833, Analog Device, Wilmington, MA, USA) and a voltage-controlled current source (VCCS). Our MCU achieves throughputs approaching 1 million instructions per second (MIPS) by executing instructions in a single clock cycle, which allows the system to optimize power consumption. The MCU was used to program the AD9833 to generate a multi-frequency sinusoidal signal through a serial peripheral interface (SPI). This signal was then converted to a 100 µA constant current by a VCCS-based Howland current pump [75]. The rationale behind choosing the current level is to obtain an optimal quality signal while ensuring user safety. Several studies [76,77,78,79] used 100 µA as the source current for measuring biological impedance; in particular, Oh et al. [80] used this current level to maximize the SNR of the measured impedance. In addition, user safety was another important consideration in determining the current level. According to the safety standard guidelines, IEC 60601-1 [81], it is limited to 100 µA for normal operation. For the VCCS, we used the high-speed amplifier (OP471, Analog Devices, Wilmington, MA, USA) featuring low noise (11 nV/Hz Max), excellent speed (8 V/ms), and a gain bandwidth of 6.5 MHz. The detailed descriptions and formulas of VCCS based on the Howland current pump are shown in Supplementary Figure S6. In our bioimpedance measurement method, we used a four-electrode method to eliminate the lead and contact resistance to perform a more reliable measurement. When a constant sinusoidal current flows through the selected CS electrode pair, the voltage sensed at the vs. electrode pair was amplified by a differential amplifier (G). However, even with the four-electrode method, measurement error may occur when the input impedance of the voltage sensing unit is not sufficiently large. An ideal voltage sensing unit should have an input impedance of infinity, and no current should flow in the signal path of the voltage electrode to accurately measure the voltage. A decrease in the input impedance of the voltage sensing unit may reduce the resolution of the measured voltage values, thereby causing a loss of feature information that distinguishes an individual, thereby causing performance degradation. Therefore, we used an instrumentation amplifier (INA128, Texas Instruments, Dallas, TX, USA) that provides a very high input impedance (10^10^ Ω) with 120 dB of common-mode rejection ratio (CMRR). It has been outfitted with input protection circuit and input buffer amplifiers, which eliminate the need for input impedance matching and make the amplifier particularly suitable for use in measurement. The system is designed to sequentially measure the impedance of 10 electrode combinations by electrically switching the five electrode pairs mounted on the device using a dual single-pole quad-throw analog switch (CD4052, Texas Instruments, Dallas, TX, USA) as a multiplexer/demultiplexer. This CMOS Single 8-Channel Analog multiplexer/demultiplexer provides a very low OFF leakage current and dissipates extremely low quiescent power over the supply voltage ranges. Through this switching operation, the impedance detection circuit lines are connected to two electrode pairs (electrode pairs of current sourcing and voltage sensing) by selecting two out of five electrodes in sequence. The measurement frequency range is from 20 kHz to 500 kHz with 20 kHz steps, and total measurement requires 7.25 s. Regarding the use of frequencies, ideally the impedance value is, in principle, not influenced by skin resistance in our four-electrode method; however, there was a slight change in impedance especially below 20 kHz experimentally. We speculate that this was because the effect of imperfect matching of resistors in Howland current pump contributes more in lower frequency ranges. Therefore, to minimize these effects, data were acquired at frequencies above 20 kHz. Additionally, we determined the upper limit of the high frequency range based on previous studies on the constituent measurement using body impedance [82,83,84,85], which reported that a 500 kHz frequency is sufficiently high for the estimation of total body water, including intracellular fluid (ICF). The electrodes we designed were plated with chrome, mounted on the top and bottom of the device, and electrically connected to the PCB layer inside the case. To keep the contact area of the finger electrode to each subject constant, the size of the electrode was designed to be 0.8 × 0.5 cm^2^, which is about 50 to 70% of the contact area with a general finger. The measured voltage is converted to a DC signal by an RMS to DC converter (AD536AKD, Analog Devices, Wilmington, MA, USA) and then obtained through AD conversion. The RMS–DC converter directly computes the true RMS of the measured voltages and provides an equivalent DC output, which is well suited for a wide variety of battery-powered wearables applications. In order to improve the repeatability of the measurement, each finger electrode area is designed in a concave shape to position the finger. The acquired impedance data is transmitted from the microcontroller to a PC via wireless communication using a Bluetooth module (BlueMod+SR, Telit, London, UK) which has a very small form factor (17 × 10 × 2.6 mm^3^), providing a dual mode Bluetooth 4.0 module. The device is powered by a lithium polymer battery (3.7 V, 200 mAh) which is rechargeable by an integrated circuit (IC). To investigate the measurement accuracy and repeatability in terms of the operating characteristics of the system, a register of 100 Ω was connected to the load of our system, and impedance measurements were performed, and it showed less than 0.0019% noise for accurate and repeatable measurements (<1.9 mΩ, 100 Ω at 50 kHz, 25 measurements). In terms of dynamic response characteristics, the response time is 7.5 µs and provides unity-gain stability within our measurement frequency range up to 500 kHz. Further details on the operational stability of the measurement system against environmental temperature and humidity changes (Supplementary Figure S7) are included in the Supplementary Materials. For device power consumption, the independent current consumption of the main circuit of our device is 10.5 mA, and 16 mA is additionally consumed during Bluetooth data transmission, resulting in a total current consumption of 26.5 mA. During continuous measurement, it can be used for about 7.5 h with a 200 mAh battery. In the actual use environment, it is sufficient to only perform authentication occasionally, so the usage time can be increased by switching the device to idle mode.

### 4.2. Human Measurement of Wearable Electrical Impedance Spectrum

The study was approved by the Institutional Review Board of the Ministry of Health and Welfare of the Republic of Korea, and written consent was obtained from all study participants. All experiments were conducted in accordance with relevant guidelines and regulations. The experiments were conducted from February to June 2021 in the designated laboratory room of our research institute with 33 subjects. Subjects visited once a day at weekly intervals for 5 weeks and participated in a total of 5 independent days, providing 8–10 measurements per session within 1 h per day. Subjects placed the device on their left wrist and their right fingers pressed the electrodes mounted on the upper side of the device. We measured the impedance for the frequency range from 20 kHz to 500 kHz with 20 kHz intervals. In order to analyze the impedance spectrum of different electrode pairs, principal component analysis was performed for each upper limb and finger impedance spectra using MATLAB (R2020b, MathWorks, Natick, MA, USA).

### 4.3. Upper Limb Impedance Features

In order to exclude the finger impedance from the measurement data, we devised a method for extracting the upper limb impedance, as shown in Figure 2. A total of 10 measurable electrode combinations can be obtained by sequentially selecting two of five electrodes mounted on the device. Of the 10 measurable electrode combinations, 6 finger impedance pairs, (*e*_1_, *e*_2_), (*e*_1_, *e*_3_), (*e*_1_, *e*_4_), (*e*_2_, *e*_3_), (*e*_2_, *e*_4_) and (*e*_3_, *e*_4_), can be obtained from the measurement of the right fingers at the electrodes mounted on the upper side of the device, and the impedance including the upper limb can be obtained from 4 combinations, (*e*_0_, *e*_1_), (*e*_0_, *e*_2_), (*e*_0_, *e*_3_) and (*e*_0_, *e*_4_), which include *e*_0_ for wrist contact. Here, the pure upper limb impedance can be obtained by removing the finger impedance measurement value included in the calculation from the summation of the measurement combination values including the electrode *e*_0_ by the mechanism described in Figure 2. Through this mechanism, there are a total of 6 combinations for obtaining upper limb impedance in this device measurement. These combinations are obtained by selecting an additional two electrodes to be included in the calculation along with the *e*_0_ electrode ((*e*_0_, *e*_1_, *e*_2_), (*e*_0_, *e*_1_, *e*_3_), (*e*_0_, *e*_1_, *e*_4_), (*e*_0_, *e*_2_, *e*_3_), (*e*_0_, *e*_2_, *e*_4_), (*e*_0_, *e*_3_, *e*_4_)). Then, each feature vector (25, 1) of the six extracted combinations was sequentially concatenated, fused into a single feature vector (150, 1), and then fed into each machine learning classifier. For our 1-D CNN model, six combinations of upper limb feature vectors were used as each channel of the input data and fed into 1D (25 × 1 × 6) forms.

### 4.4. Ratiometric Features

In this study, the term for the ratiometric method, as proposed in our previous study [39], is the recorded value of a particular finger pair divided by the value of another finger pair obtained from the same measurement at the same frequency. The measurement using our device could extract 15 possible combinations of finger pairs by selecting two out of six finger electrode combinations ((*e*_1_, *e*_2_), (*e*_1_, *e*_3_), (*e*_1_, *e*_4_), (*e*_2_, *e*_3_), (*e*_2_, *e*_4_), (*e*_3_, *e*_4_)). For the ratiometric features in this study, a total of 6 combinations were selected so that the combination of finger pairs in the calculation uses each electrode pair only once for the numerator and denominator as in our previous study [36]. The selected 6 ratiometric features were (*e*_1_, *e*_2_)/(*e*_3_, *e*_4_), (*e*_1_, *e*_3_)/(*e*_1_, *e*_2_), (*e*_1_, *e*_4_)/(*e*_1_, *e*_3_), (*e*_2_, *e*_3_)/(*e*_1_, *e*_4_), (*e*_2_, *e*_4_)/(*e*_2_, *e*_3_) and (*e*_3_, *e*_4_)/(*e*_2_, *e*_4_), which contains a total of 150 samples, the same number as both finger impedance and upper limb features.

### 4.5. Recognition of Individuals

For individual identification, the most commonly used machine learning classification models of RF, kNN, LDA and SVM were applied and implemented in MATLAB using an error-correcting output code for multi-class classification [86]. We used an RF classifier, a machine learning model that achieved the highest classification performance in our previous finger acoustic transmission-based biometric authentication study [86]. Our RF classifier consisted of 150 trees, and we used the Gini diversity index for the splitting criterion. The maximum number of decision splits was set to 150, the minimum number of leaves was set to 1, and others were set to default values. For the LDA classification model, which does not require parameter adjustment and provides a computationally efficient operation [87,88], we used the default setting in MATLAB. The kNN classifier, which showed the highest accuracy in our previous study [39], that proposed the impedance-based ratiometric method was also tested. The KNN model assigns weights so that nearer neighbors contribute more to the average, and the neighbors are taken from the corresponding set of objects in the class [89]. The nearest neighbor number was set to 1 after parametric searching from 1 to 20, and the Euclidean distance metric was used. The SVM, one of the most popular machine learning algorithms, was also tested. We used the second polynomial kernel function, and the tuning parameter C was set to 1.0. The quadratic kernel function was chosen because it obtained better performance than the linear kernel in our previous work [39]. The hyperparameters were selected using the ‘Optimize Hyperparameters’ option in MATLAB.

For deep learning analysis, we applied the modified SB-CNN [90] algorithm used in our previous study for individual classification based on finger acoustic transmission characteristics. CNN is well known as a deep neural network designed for image analysis, and recently it has been reported that CNN has excellent capacity in continuous data as well [91]. In this study, each combination of impedance-based features was a one-dimensional signal vector consisting of 25 samples, which were used as each channel of 1D (25 × 1 × channel) inputs for 1D CNN analysis. The CNN model we used has three convolutional layers, two max-pooling layers, followed by two fully connected layers. The parameters of the CNN architecture have the following detailed architecture with the input size of (25 × 1 × channel). The first two convolutional layers were followed by max-pooling with a stride of the same size as the pooling dimensions, which reduced the dimensions of the spatial map. The first layer *l*1 consisted of 24 kernels with an accept field of (5, 1), with padding (i.e., the same padding) to reserve the size of the input data. This was followed by batch normalization and the activation function of rectified linear units (ReLU), h(x) = max (x, 0) max-pooling with stride (2, 1). The second layer *l*2 has 48 kernels with an acceptable field of (5, 1) including padding, followed by batch normalization, ReLU, and max-pooling with strides (2, 1). Then, the *l*3 layer consists of 48 kernels with receptive fields of (5, 1) with padding, batch normalization, and ReLU, which are followed by fully connected (flattened) layers and an output layer with 33 units by a softmax activation. In the CNN model, Adam Optimizer was chosen, and the constant learning rate was set to 0.0001. The mini-batch size was set to 128, and each dataset was trained on 2000 epochs using a dropout of probability 0.5 between dense layers. The CNN algorithm was implemented using Python (version 3.7.4) with Keras (version 2.2.4) and tensorflow (version 2.2.0) on a Quadro RTX 8000 D6 48GB GPU (NVIDIA, Santa Clara, CA, USA).

To validate classification accuracy, each classifier was evaluated using n-fold (n: number of measurement days) cross validation for 1627 datasets from 33 subjects. For robust classification, the data were divided into five equal-sized subgroups by indexing the data from 1 to 5 according to each subject’s measurement date so that each subgroup contained mutually exclusive data on the same date, and n-fold cross validation was performed. Through this data subgrouping, the accuracy of all classifier models was evaluated with training and test datasets, each consisting of data obtained on different dates.

The accuracy of each classifier model was evaluated to distinguish one from the others in closed-set scenarios. Here, each classifier compared obtained features with the templates of all the subjects enrolled in the database, and the identity of the person whose template has the highest degree of similarity with the obtained data was assigned to the class. Additionally, the sensitivity and specificity for the ROC curves were evaluated by changing the threshold of posterior probability to assess the ability to recognize and reject the unregistered users who present inputs. Then, we evaluated the area under the ROC curve (AUC) and the equal error rate (EER) where FAR equals FRR.

## Figures and Tables

**Table 1:** Accuracy, EER, and AUC of discriminative classification models according to feature selections.

		Accuracy (%)				EER (%)				AUC			
	Feature	Finger	Ratio	u-Limb	Ratio + u-Limb	Finger	Ratio	u-Limb	Ratio + u-Limb	Finger	Ratio	u-Limb	Ratio + u-Limb
Classifier													
RF		70.83	88.21	89.98	96.43	7.5460	2.1570	3.0275	0.6050	0.9793	0.9974	0.9968	0.9998
LDA		72.53	88.75	87.52	96.00	6.6380	3.0731	3.0117	1.3022	0.9826	0.9930	0.9947	0.9967
kNN		60.66	87.46	74.86	96.31	20.2827	6.4651	12.9619	1.9015	0.7972	0.9353	0.8704	0.9810
SVM		63.55	85.74	75.29	92.99	12.7228	3.7646	6.2097	2.7668	0.9500	0.9938	0.9872	0.9970
CNN		77.62	94.40	95.70	97.60	5.0524	1.1063	0.9834	0.4917	0.9891	0.9990	0.9993	0.9999

## Data Availability

The data presented in this study are available from the corresponding author on reasonable request.

## Supplementary Materials

The following are available online at www.mdpi.com/article/10.3390/bios11100398/s1. Figure S1: Comparison of temporal variation in upper limb including finger impedance (before finger removal) and pure upper limb (after finger removal) of three subjects measured over three independent days, Figure S2: Enhanced ratiometric features reducing undesirable variation, Figure S3: The effect of finger placement changes on the ratiometric feature, Figure S4: 1D-CNN model consisting of three convolution layers, two pooling layers, and two fully connected layers, Figure S5: The boxplots of the accuracy according to the number of subjects, Figure S6: The voltage-controlled current source based on Howland current pump, Figure S7: Comparison of impedance change in two-electrode measurement method and four-electrode measurement method according to the effect of moisture.
