# Supplementary material for: Electrical Impedance of Upper Limb Enables Robust Wearable Identity Recognition against Variation in Finger Placement and Environmental Factors
Source: Biosensors (Basel). 2021 Oct 16;11(10):398. doi: 10.3390/bios11100398 (PMC8534261; doi:10.3390/bios11100398)
Supplement: Supplementary file 1 [file biosensors-11-00398-s001.zip › biosensors-1403744-supplementary.pdf]

Supplementary Materials for

Electrical Impedance of Upper Limb Enables Robust Wearable Identity Recognition against Variation in Finger Placement and Environmental Factors

Hyung Wook Noh [1,2,†], Joo Yong Sim [3,†], Chang-Geun Ahn [1], Yunseo Ku [2,*]

[1] Bio-Medical IT Convergence Research Department, Electronics and Telecommunications Research Institute, Daejeon 34129, Korea; happy05@etri.re.kr (H.W.N.); cgahn@etri.re.kr (C.-G.A.)

[2] Department of Biomedical Engineering, College of Medicine, Chungnam National University, Daejeon 34134, Republic of Korea

[3] Department of Mechanical Systems Engineering Department, Sookmyung Women's University, Seoul 04310, Republic of Korea; jysim@sookmyung.ac.kr (J.Y.S.)

* Correspondence: yunseo.ku@cnu.ac.kr

† These authors contributed equally to this study.

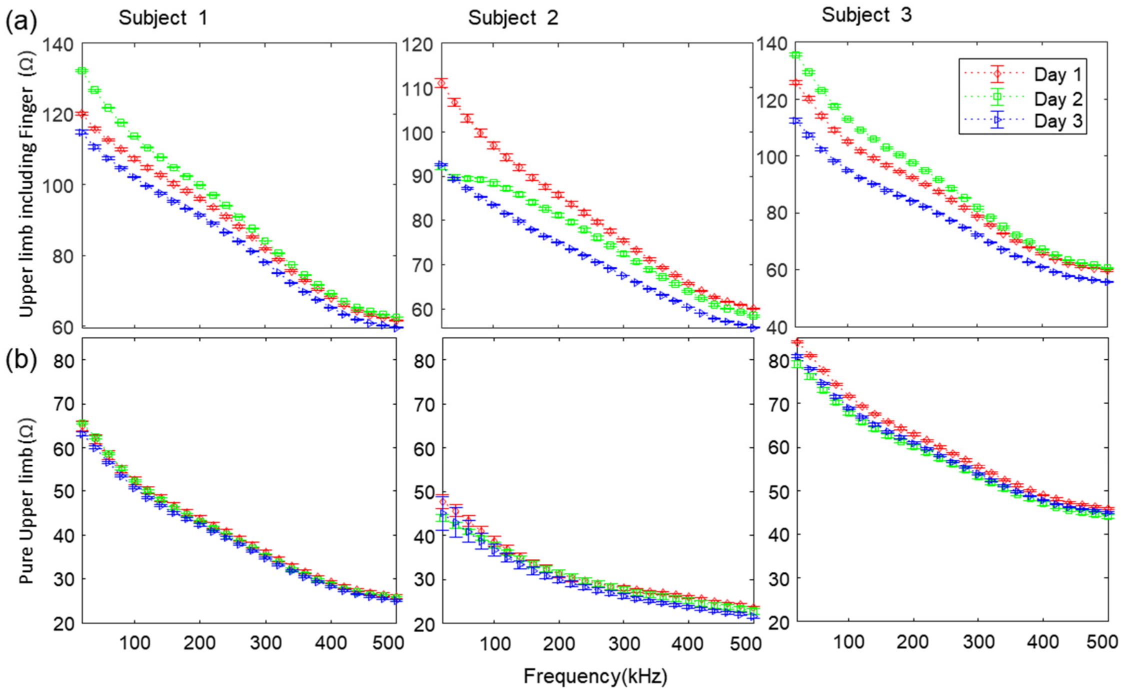

Supplementary Figure S1. Comparison of temporal variation in upper limb including finger impedance (before finger removal) and pure upper limb (after finger removal) of 3 subjects measured over three independent days. (a) The impedance before finger removal. (b) The impedance after finger removal (pure upper limb). The error bars in the figure indicate the relative standard deviation. (n = 10).

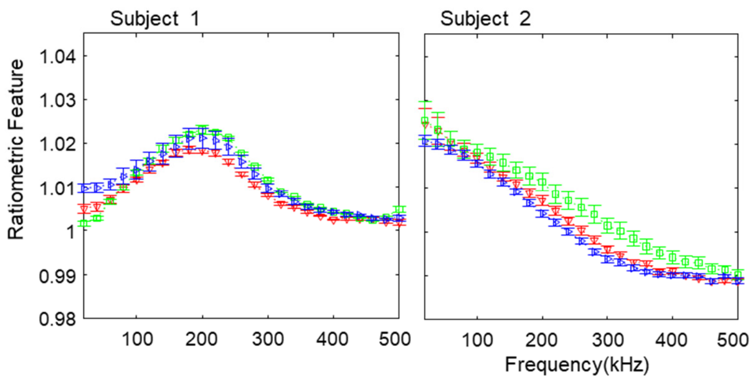

Supplementary Figure S2. Enhanced ratiometric features reducing undesirable variation. Variation of the ratiometric features for three subjects measured for independent three days. Values are the mean ± S.D. (n = 10).

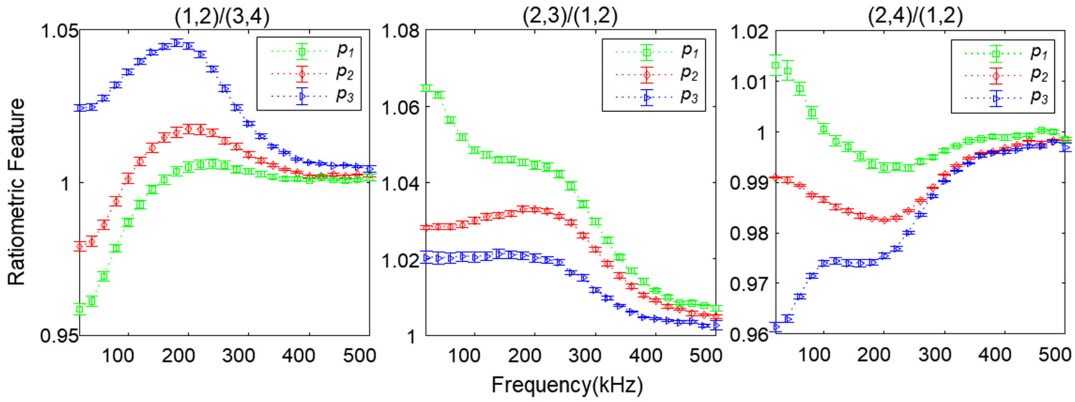

Supplementary Figure S3. The effect of finger placement changes on the ratiometric feature. Values are the mean ± S.D. (n = 10).

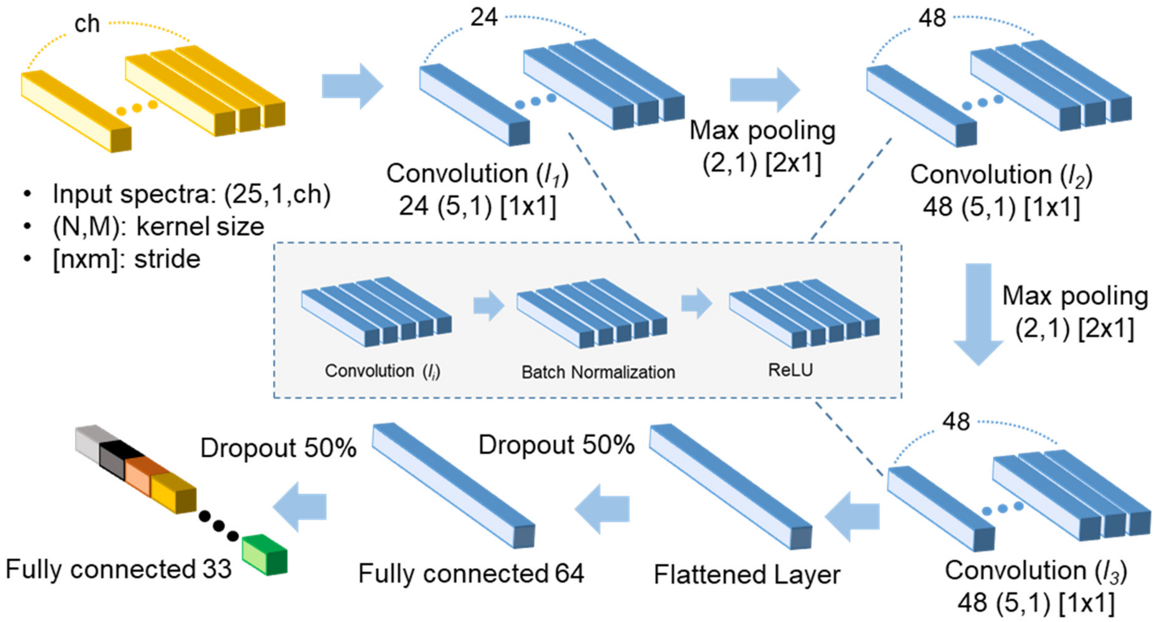

Supplementary Figure S4. 1D-CNN model consisting of three convolution layers, two pooling layers, and two fully connected layers. Each feature vector of impedance data consisting of 25 samples formed into a respective channel in a 1-D input (25 × 1 × channel) and fed into the model.

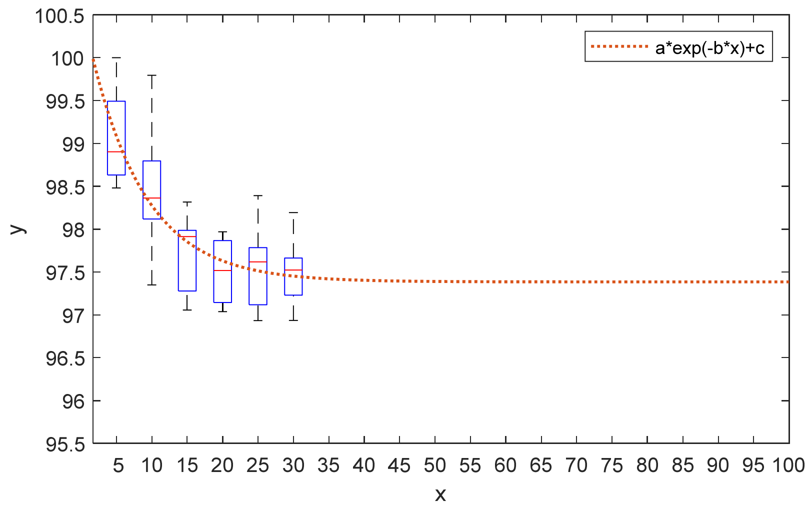

Supplementary Figure S5. The boxplots of the accuracy according to the number of subjects. Each classification accuracy for the selected dataset was acquired using the RF model. The green dotted line represents the accuracy prediction curve as the number of subjects increases. The inset equation's parameters were 3.24, 0.1289, 97.38 for a, b, and c, respectively.

Howland Current Pump for the VCCS Implementation

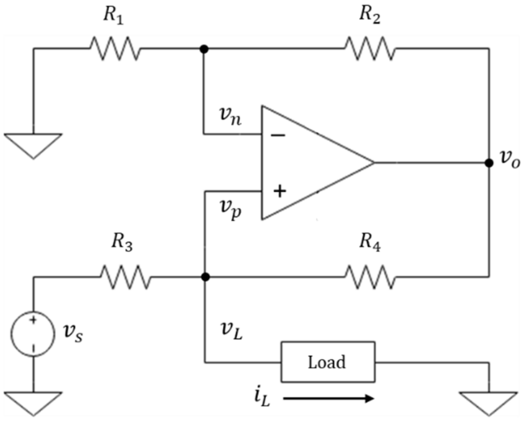

Supplementary Figure S6. The voltage-controlled current source based on Howland current pump [1].

Due to a virtual short circuit, following equation (1) is satisfied as below,

$$\frac{v_n}{R_1} = \frac{v_n - v_o}{R_2} = 0 \tag{1}$$

Then, equation (2) can be rewritten as follows,

$$\left(\frac{1}{R_1} + \frac{1}{R_2}\right)v_n = \frac{v_o}{R_2} \tag{2}$$

Solving the v_n, the following equation (3) can be obtained.

$$v_n = v_p = v_L = \frac{\frac{1}{R_2}}{\frac{1}{R_1}+\frac{1}{R_2}} v_o = \frac{1}{R_1+R_2} v_o \tag{3}$$

The load current i_L is given by following equation (4) as below,

$$i_L = \frac{v_n - v_o}{R_3} + \frac{v_n - v_o}{R_4} = \frac{v_n - v_o}{R_3} + \frac{\frac{R_1+R_2}{R_1} v_L - v_L}{R_4} = \frac{v_s}{R_3} - \left(\frac{1}{R_3} + \frac{R_2}{R_1 R_4}\right) v_L$$

$$= \frac{v_s}{R_3} - \frac{R_1 R_4 - R_2 R_3}{R_1 R_3 R_4} v_L = \frac{v_s}{R_3} - \frac{v_L}{\frac{R_4}{\frac{1}{R_1}+\frac{1}{R_2}}} \tag{4}$$

Here, the output impedance Z_{out} is defined as following equation (5).

$$Z_{out} = \frac{R_4}{\frac{R_4}{R_3} - \frac{R_2}{R_1}} \tag{5}$$

Then, the equation (4) becomes as follows,

$$i_L = \frac{v_s}{R_3} - \frac{v_L}{Z_{out}} \tag{6}$$

If the following equation (7) is satisfied, output impedance Z_{out} becomes infinity.

$$\frac{R_4}{R_3} = \frac{R_2}{R_1} \tag{7}$$

Thus, the load current i_L is given as follows,

$$i_L = \frac{v_s}{R_3} \tag{8}$$

Therefore, the load current i_L (8) does not depend on the load, but only depends on the input voltage v_s.

Operational stability against environmental temperature and humidity changes

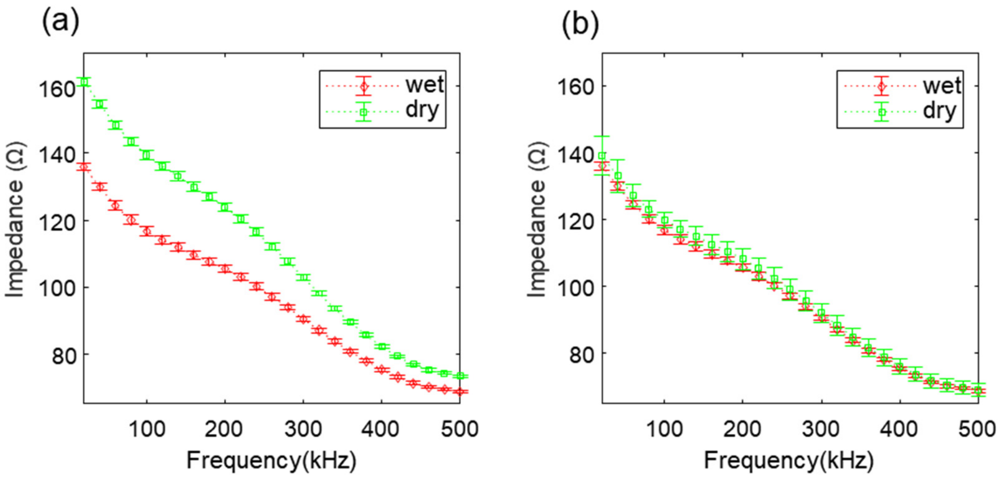

Supplementary Figure S7. Comparison of impedance change in two-electrode measurement method and four-electrode measurement method according to the effect of moisture. (a) Impedance change according to the effect of moisture in the two-electrode measurement method. (b) Impedance change according to the effect of moisture in the four-electrode measurement method.

For the electrical components used in our system, including the current source (CS) and the voltage sensing unit (VS), temperature or humidity variations within the operating range can cause changes in electrical properties, which could affect reproducibility and accuracy. In our study to evaluate the variability of impedance measurement data for external temperature changes, we changed the external temperature in the range of 18°C to 32°C degrees. The variability of the obtained data corresponding to each temperature changes was analyzed, and it was verified that the upper limb impedance was relatively robust than the finger impedance, which can be used as a more salient feature for improving biometric authentication. According to relative literature, most commercial electronic devices are generally set to operate in a temperature range of 0°C to 70°C [2]. To investigate the normal operating temperature range of our system, we reviewed all data sheets of electronic components used in our wearable devices, and the common operating temperature range for all components to ensure reliable performance is -40°C to 85°C. However, despite our device's operating temperature range according to the datasheet of each component is wide enough for commercial use, the performance may decrease in actual application environments. An indirect evaluation of the system's wider operating temperature ranges referenced here may require further experimentation for better representation. Therefore, in future work, we will validate our method in a wider range of temperature to provide direct evidence.

In addition, even under the influence of external humidity, the system is required to maintain the stability of the signal quality in both low and high ambient humidity. However, changes in environmental humidity can affect skin electrode contact resistance. The increase of free water content of the electrode or the water content in the air around the electrode may also increase the water content of the dielectric and reduce the skin–electrode contact resistance [3]. These changes in humidity can cause reduced reproducibility through unstable data acquisition. To overcome this problem, we applied a four-electrode method in our system. The applied four-electrode method removes the lead and skin electrode contact resistance by separating the current and voltage electrodes [4,5]. To investigate this effect, we observed the change in finger impedance by changing the relative humidity (RH) of the finger in contact with the electrode in our four-electrode method and our designed two-electrode method-based measurement platform, respectively. First, the finger impedance in a dry state (24.5% RH) was measured, then the fingertip was lightly moistened with water and measured in a wet state (56.2% RH). The finger temperature was kept constant (36.7°C) in each state. The data were measured 10 times per each state and displayed as mean and standard deviation. The two-electrode method results showed that the measured data of two different states of fingertip moisture (wet, dry) showed significant impedance changes as shown in Supplementary Figure S7 (a). In contrast, the data measured by the four-electrode method showed that the features obtained in the two different states were stable and reproducible, as shown in Supplementary Figure S7 (b). The experimental results indicate that the obtained data based on our four-electrode method is reliable regardless of the influence of external humidity.

References

1. Franco, S. Design with Operational Amplifiers and Analog Integrated Circuits; McGraw-Hill series in electrical and computer engineering; 3rd ed.; McGraw-Hill: New York, 2002; ISBN 978-0-07-232084-8.
2. Hardware and Computer Organization - 1st Edition Available online: https://www.elsevier.com/books/hardware-and-computer-organization/berger/978-0-7506-7886-5 (accessed on 2 October 2021).

3. Tang, Y.; Chang, R.; Zhang, L.; Yan, F.; Ma, H.; Bu, X. Electrode Humidification Design for Artifact Reduction in Capacitive ECG Measurements. Sensors 2020, 20, 3449.
4. Zhang, Z.; Rouabhia, M.; Moulton, S.E. Conductive Polymers: Electrical Interactions in Cell Biology and Medicine; CRC Press, 2018; ISBN 978-1-4822-5931-5.
5. Hoffer, E.C.; Meador, C.K.; Simpson, D.C. Correlation of Whole-Body Impedance with Total Body Water Volume. J. Appl. Physiol. 1969, 27, 531–534.
